# Smartwatch-Derived VO2max Prediction Model for Korean Adults: Utilizing Heart Rate and GPS Data from the 12-Minute Cooper Test

**DOI:** 10.3390/healthcare13070722

**Published:** 2025-03-25

**Authors:** Kihyuk Lee, Dohee Kim, Sungeun Shin, Hongjun Choi, Ahyun Jang, Jinwook Chung

**Affiliations:** 1 Data Convergence Team, Seoul National University Bundang Hospital, Seongnam 13605, Republic of Korea; lkhlike@naver.com; 2 Department of Sports Science Convergence, College of Arts, Dongguk University, Seoul 04620, Republic of Korea; tara.kim1171@gmail.com (D.K.); mysoulmate1026@gmail.com (S.S.); hongjun0802@naver.com (H.C.); ahhhhhjji@naver.com (A.J.)

**Keywords:** VO2max, prediction model, Cooper test, smartwatch, running

## Abstract

**Background/Objectives**: Recent technological advancements enable smartwatches to measure running distance and heart rate using wearable sensors. This study aimed to analyze the validity of the 12 min Cooper test using a smartwatch and to develop an accurate VO2max prediction model for Korean adults. **Methods**: A total of 104 adults (53 males: age 35.00 ± 6.1 years, BMI 24.71 ± 3.13; 51 females: age 34.82 ± 6.07 years, BMI 22.24 ± 2.66) participated. VO2max was measured using a maximal graded treadmill test. Participants performed the Cooper test while wearing a smartwatch, which collected average heart rate, peak heart rate, and running distance. Sex, height, and weight were also included as predictor variables. Multiple regression analysis was conducted to develop a VO2max prediction equation. Model accuracy was assessed using R^2^ and the standard error of the estimate (SEE). **Results**: The developed VO2max prediction equation was VO2max = 27.620 + 6.358 (sex; male = 1, female = 0) − 0.012 (height) − 0.202 (weight) − 0.036 (mean HR) + 0.039 (peak HR) + 0.012 (distance) (R^2^ = 0.853, SEE = 3.176 mL/kg/min, *p* < 0.001). The intra-class correlation coefficient (ICC) between measured and predicted VO2max using the smartwatch was 0.961, compared to 0.925 for traditional methods. The explanatory power was 86.0% (SEE = 3.024 mL/kg/min) versus 81.0% (SEE = 3.516 mL/kg/min). **Conclusions**: The smartwatch-based VO2max prediction model demonstrated higher accuracy than traditional methods. This equation is recommended for more precise VO2max estimation in Korean adults.

## 1. Introduction

Aerobic capacity refers to the ability of the circulatory and respiratory systems to utilize oxygen efficiently. Maximum oxygen uptake (VO2max) is one of the key indicators that can quantify aerobic capacity [1,2]. A high VO2max signifies that the body can use greater amounts of oxygen during physical activity, allowing for increased energy production [3]. This not only improves aerobic exercise performance but also aids in preventing cardiovascular diseases and promoting overall health [4].

VO2max is most accurately measured in a laboratory setting by directly analyzing oxygen consumption during a maximal graded exercise test (GXT) using a bicycle ergometer or treadmill along with respiratory gas analysis [5,6]. However, this method requires complex experimental protocols, specialized equipment, and a controlled laboratory environment, which limits its practical application [7]. To address these limitations, field tests and estimation formulas have been developed to indirectly estimate VO2max in the field [8]. Cooper developed a field test in which participants walk or run as far as possible within 12 min on a 400 m track to estimate VO2max [9]. In a study involving 105 participants, Cooper reported a strong positive correlation (r = 0.897) between the 12 min running distance and VO2max and derived an equation for estimating VO2max using linear regression analysis [9].

The 12 min Cooper test (Cooper test) developed by Cooper has been used in various fields, such as designing training programs for elite athletes and researching cardiorespiratory endurance to assess aerobic capacity [10,11,12]. However, compared to studies that have developed VO2max estimation equations using other field tests, there have been limited studies on the estimation and validation of VO2max. Most studies that use field tests to estimate VO2max have focused on the 20 m shuttle run and step tests, and these studies span a wide range of age groups and ethnicities [8,13]. On the other hand, studies on VO2max estimation equations using the Cooper test have been limited to male participants [9,14]. Furthermore, the Cooper test has not been cross-validated to verify the accuracy of its estimation equation, and variables other than running distance that may influence VO2max have not been considered.

Meanwhile, with advancements in technology, it has become possible to measure running distance and heart rate using Global Positioning System (GPS) and photoplethysmogram (PPG) sensors integrated into wearable devices. Various studies have utilized these technologies [15]. One study validated a method for estimating energy expenditure during running using a smartwatch [16], while another verified the accuracy of estimated oxygen consumption through a smartwatch [17,18]. Furthermore, wearable devices have made significant advancements in cardiovascular health monitoring by providing real-time data on heart rate, blood pressure, and ECG, enabling early diagnosis and prevention of cardiovascular diseases [19]. Similarly, in sports diagnostics, these devices allow for continuous assessment of athletic performance, including VO2max and muscle recovery, contributing to the optimization of training programs and the reduction of injury risk [19]. These findings suggest that combining smartwatch measurements with the Cooper test may provide a practical and accurate method for estimating VO2max in real-world settings. Therefore, the purpose of this study was to develop a predictive model for estimating VO2max using variables measured by a smartwatch and the Cooper test, targeting adult men and women in South Korea, and to validate the accuracy of this model by comparing it with existing estimation equations.

## 2. Materials and Methods

### 2.1. Participants

The participants in this study consisted of 104 healthy Korean adults, aged between 22 and 54 years, with no history of cardiovascular or chronic diseases. They were recruited through promotional materials provided by the academic institution. Their cardiorespiratory fitness (CRF) levels, classified based on the age- and sex-specific reference values proposed by McKay et al. (2022), fell within tiers from 0 to 2 [20]. This classification provides a standardized approach for interpreting VO2max values in the general adult population.

Before this study began, participants were informed about the planned measurements, and written consent was obtained. This study was approved by the Institutional Review Board (IRB) of Dongguk University (DUIRB-2024-04-02). To ensure data accuracy, individuals with conditions that could affect physical activity (e.g., heart disease, pulmonary disease, a history of taking antihypertensive medications, or orthopedic diseases) within the past six months were excluded from participation. Likewise, participants who were in poor condition due to overwork, alcohol consumption, or other factors the day prior were excluded from the measurement to ensure they were in optimal condition for the maximal graded exercise test (GXT) and the Cooper test. During the first visit, a physical examination was conducted. Participants’ heights and weights were measured, and they completed a survey regarding health-related habits and marathon experience. The measurements for this study were conducted between May and November. The power value and required sample size for this study were calculated using the G*Power program (version 3.1 for Windows). In this study, the effect size criterion was set to Cohen’s f^2^ = 0.15, which corresponds to a medium level of effect size according to Cohen’s (1988) guidelines [21]. In the multiple linear regression analysis, the power value was calculated to be 0.832, based on an effect size of 0.15, a sample size of 104 participants, and six predictors.

### 2.2. Procedures

The GXT and the Cooper test were conducted to develop an estimation formula based on the Cooper test with a smartwatch (Galaxy watch 7; Samsung Electronics Co., Suwon-si, Republic of Korea). To minimize interference between tests, the GXT and the Cooper tests were performed on different days, which were selected randomly. All participants were given a break of at least three days between tests to reduce the potential carryover effects of one test on the other. To ensure accurate measurements, participants were instructed to avoid excessive physical activity, smoking, and alcohol consumption on the day prior to their laboratory visit. Additionally, the consumption of stimulant-containing beverages was prohibited during testing.

### 2.3. Measurement of VO2max

For the measurement of VO2max, the GXT was performed on a treadmill (STEX-8100T; Namyangju-si, Taeha, Republic of Korea). Participants wore a wireless heart rate monitor (S610i; Polar, Bethpage, NY, USA) and rested for at least 10 min to ensure a stable condition before the measurement. VO2max was assessed using a gas analyzer (METAMAX 3B; Cortex, Leipzig, Germany). The GXT protocol followed the Bruce protocol, which is designed for adults [22]. This protocol is the most commonly used method for maximal GXT on a treadmill and has been shown in previous studies to provide reliable VO2max values, regardless of age, gender, or fitness level [23]. During the test, participants’ condition was continuously monitored using the Borg RPE 10 scale and a heart rate monitor. To ensure accurate measurement of VO2max and participant safety, the test was considered terminated when two of the following three criteria were met: (1) reaching a heart rate close to 95% of the predicted maximal heart rate (220-age), (2) no further increase in oxygen consumption despite an increasing exercise load, or (3) a change in the respiratory exchange ratio (VCO2/VO2) of at least 1.15 [24].

### 2.4. Cooper 12-Min Test

The Cooper test was conducted on a designated 400 m athletic track under the supervision of researchers [9]. Prior to the test, participants completed a 10 min warm-up consisting of continuous running at a low-to-moderate intensity. The test protocol required participants to cover the maximum possible distance within 12 min. Upon completion of the test, the distance traveled was measured using markers placed at 25 m intervals along the track, supplemented by a portable measuring wheel (ML-18MK; Komelon, Korea) for accuracy. During the test, participants wore a Samsung Galaxy Watch 7, which was used to record biometric variables and distance data via PPG and GPS tracking. The recorded biometric variables included the average heart rate (HR), the maximal HR achieved, and the running distance during the 12 min test.

### 2.5. Statistical Analysis

The analysis of all variables measured in this study was performed using the SPSS 25.0 for Windows (IBM Corp., Amonk, NY, USA). Multiple regression analysis was conducted to develop a VO2max predictive equation using height, weight, and the results measured during the Cooper test (running distance, average heart rate, and maximum heart rate) while wearing a smartwatch. The variance inflation factor (VIF) was assessed, and the VIF values ranged from 1.825 to 3.984, indicating that there were no issues with multicollinearity. Bland–Altman analysis was used to verify the agreement between the directly measured results and the predicted results, and the limit of agreement (LoA) was calculated to assess the consistency of the developed estimation equation. The LoA was calculated as the ratio of the standard error to the mean error within the calculated confidence interval (%, limit of agreement). To validate the developed prediction model, the traditional Cooper test estimation equation was also analyzed using Bland–Altman analysis. Consistency between the measured and predicted values was evaluated using the intraclass correlation coefficient (ICC) with the Two-way Random Effects Model and coefficient of variation (CV). Simple regression analysis was performed to examine the accuracy of the VO2max values predicted by the two estimations in comparison to the criterion-measured VO2max. The significance level for all statistical tests was set at 0.05.

## 3. Results

### 3.1. Results of Values from the Maximal GXT, Cooper Test with a Smartwatch, and Traditional Cooper Test

The physical characteristics of the participants are presented in Table 1. The average age of all participants was 34.91 years, with an average height of 167.70 cm, weight of 66.44 kg, BMI of 23.50 kg/m^2^, muscle mass of 28.49 kg, and body fat percentage of 22.99%. Table 2 presents values from the GXT, the Cooper test using a smartwatch, and the traditional Cooper test. The mean VO2max was 46.39 mL/kg/min based on the GXT in total, with values of 50.06 mL/kg/min for the men’s group and 42.59 mL/kg/min for the women’s group. During the GXT, the overall mean maximal heart rate (HR) was 180.45 bpm, with 182.55 bpm for men and 178.22 bpm for women. In the Cooper test using a smartwatch, the mean running distance was 2480.29 m overall, with men running 2676.42 m and women running 2276.47 m. The mean maximal HR was 183.90 bpm overall, with 185.89 bpm for the men’s group and 181.84 bpm for the women’s group. In the traditional Cooper test, the overall mean running distance was 2477.96 m, with men running 2683.32 m and women running 2264.55 m.

### 3.2. Results of Multiple Regression Model to Estimate VO2max Using a Cooper Test with a Smartwatch

Table 3 presents the results of multiple regression analysis to estimate VO2max. The VO2max regression model, calculated for both the GXT and the Cooper test using a smartwatch, showed a multiple correlation coefficient of 0.923, with an explanatory power of 85.3%. The standard error of the estimate was 3.176 mL/kg/min. Table 4 provides the formula for estimating maximal oxygen uptake using both the Cooper test with a smartwatch and the traditional Cooper test.

### 3.3. Comparison of the Validity of the Cooper Test Using a Smartwatch vs. a Traditional Cooper Test

**Figure 1:**
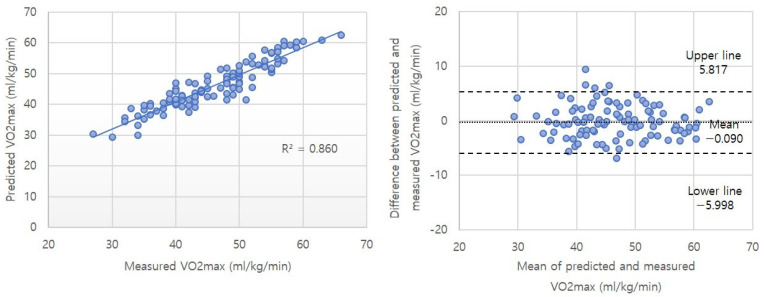
Correlation analysis and Bland–Altman limit of agreement between predicted and measured VO2max values in the Cooper test with a smartwatch.

**Figure 2:**
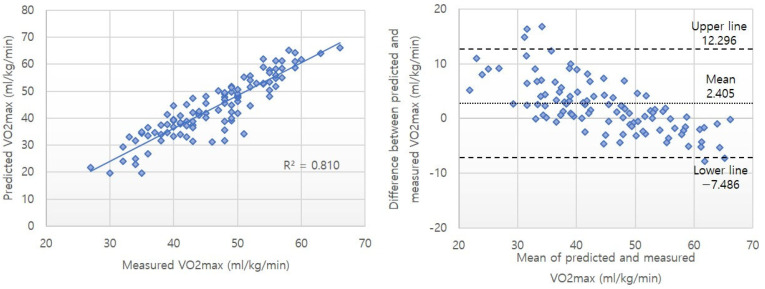
Correlation analysis and Bland–Altman limit of agreement between predicted and measured VO2max values in the traditional Cooper test.

The differences between the measured VO2max and the predicted VO2max were −0.09 ± 3.01 and 2.40 ± 5.05 mL/kg/min for the Cooper test using a smartwatch and the traditional Cooper test, respectively (Table 4). The intra-class correlation (ICC) between the measured VO2max and the predicted VO2max was 0.961 and 0.925 in the Cooper test using a smartwatch and the traditional Cooper test, respectively. The coefficient of variation (CV) values were 16.39% and 24.69%. Simple regression analysis results indicated significant relationships between the criterion-measured and predicted VO2max in the Cooper test using a smartwatch and the traditional Cooper test. The R^2^ values were 0.860 and 0.810 for the Cooper test using a smartwatch and the traditional Cooper test, respectively, and the standard errors of estimate (SEE) were 3.024 and 3.516 mL/kg/min (Table 5). Figure 1 and Figure 2 shows the correlation analysis and Bland–Altman limit of agreement between predicted and measured VO2max values.

## 4. Discussion

Previous studies on the Cooper test have primarily focused on analyzing the correlation between aerobic capacity and running distance, particularly in males or adolescents [25,26]. Furthermore, studies involving Asian populations in relation to the Cooper test remain insufficient. Therefore, it is essential to conduct studies targeting Asian men and women across various age groups. The purpose of this study was to develop an accurate VO2max predictive model using the 12 min Cooper test with a smartwatch in Korean adults. In our study, the predictors for the estimation equation were gender, height, weight, mean HR, maximal HR, and running distance (R^2^ = 0.923, SEE = 3.176 mL/kg/min, *p* < 0.001).

These predictor variables differed from those used in previous studies that developed VO2max prediction models based on the 12 min Cooper test. In a previous study, Cooper et al. developed an estimation formula using only running distance from the Cooper test [9]. In this study, the correlation between VO2max and running distance was 0.897 for men. However, this study included only 115 men aged from 17 to 52 years. Another previous study examined 80 boys aged from 11 to 14 years using the 12 min Cooper test and reported a correlation of 0.650 between aerobic capacity and running distance [27]. However, both studies exclusively focused on male participants [9,27]. Most studies on the Cooper test to date have primarily analyzed the correlation between aerobic capacity and running distance in males or adolescents [9,25,26,27]. Meanwhile, Penry et al. investigated the validity and reliability of the Cooper test and the 20m shuttle run among 60 men and women aged from 18 to 33 years [28]. They found a high correlation (r = 0.86) between the results of the incremental treadmill run with expired gas analysis and Cooper test performance. However, they reported that the Cooper test systematically underestimated reference scores at lower values and overestimated them at higher values for both men and women [28].

Unlike previous studies, our study developed a more reliable VO2max prediction model using heart rate and running distance measured with a smartwatch among 104 adults aged from 23 to 54. Validation of the developed VO2max prediction model revealed a significant correlation between the predicted VO2max values and the actual measured VO2max values (r = 0.927, SEE = 3.024 mL/kg/min). In comparison, the correlation between VO2max values predicted using the traditional Cooper test equation and the actual measured VO2max values was lower than that of the prediction model developed in this study (r = 0.900, SEE = 3.516 mL/kg/min).

To verify the applicability of the developed prediction model, the Bland–Altman limit of agreement analysis [29] was applied. The difference between the predicted VO2max values using the smartphone-based model and the actual measured values was −0.09 ± 3.01 mL/kg/min, while the difference between the values from the traditional method was 2.405 ± 5.05 mL/kg/min. The results from our newly developed prediction model were similar to those of a previous study that developed a VO2max prediction model for male college students using the Cooper test (from −0.31 to 2.09 mL/kg/min). Furthermore, these results were comparable to the differences observed in previous prediction models developed using the treadmill test, YMCA step test, and shuttle run test (0.108 ± 4.53 mL/kg/min, 0.020 ± 4.46 mL/kg/min, −0.010 ± 3.71 mL/kg/min, respectively). This suggests that the developed prediction model is well-aligned with the actual measured values. The Bland–Altman plot (Figure 1) visually demonstrates the agreement between the two methods, showing that most data points are within the 95% limits of agreement. Moreover, applying the traditional Cooper test equation to the results of this study and comparing it with our developed prediction model demonstrated that our model had superior explanatory power (R^2^ = 0.860 vs. 0.810).

The main result of our study was the development of a significant VO2max prediction model for Korean adults using the 12 min Cooper test. Furthermore, we developed a VO2max prediction model that is more accurate and reliable than the traditional 12 min Cooper test estimation formula. This was achieved solely by wearing a smartwatch, without the need to measure actual running distance. The developed VO2max prediction model can be particularly useful for patients with cardiovascular diseases, especially those with heart failure. As it is smartphone-based and easily accessible, it can also be applied for everyday health management and exercise assessment. Peak VO2 in heart failure patients is a critical physiological indicator, essential not only for assessing exercise capacity but also for evaluating cardiovascular status. This model can estimate an individual’s VO2max and provide personalized exercise prescriptions accordingly. Furthermore, by using the smartphone to estimate VO2max and create an exercise log, fitness improvement trends can be monitored. When integrated into a telemedicine system, this model allows for remote tracking and management of a patient’s condition. A previous study by Tedeschi et al. (2024) has shown that peak VO2 evaluation is a significant indicator of prognosis in heart failure patients, further supporting the relevance of this model [30]. This tool can be an important resource for the general public to continuously manage their exercise capacity and improve cardiovascular health.

However, there were some limitations to this study. First, the participants were Korean adults aged from 23 to 54, and changes in VO2max according to age were not considered. Second, caution should be exercised when applying this prediction model to other racial groups. Race is one of the important factors that explain the differences in VO2max [31]. To develop a more precise prediction model, age-related factors should be considered, and validation across different racial groups is necessary. Third, the regression model employed in this study assumes a linear relationship and is tailored to the specific dataset, which may lead to reduced predictive performance when applied to new data. Furthermore, a previous study has indicated that recalibrating predictive models can improve their accuracy [32]. Therefore, future research should focus on expanding the validation sample and exploring the use of various modeling techniques to enhance the generalizability and precision of the model.

## 5. Conclusions

We developed and validated a VO2max prediction model for Korean adults using the GXT and the 12 min Cooper test conducted via a smartwatch. The VO2max values predicted by the prediction model developed in this study showed a significant correlation with the directly measured VO2max values. Furthermore, the prediction model demonstrated greater explanatory power and a lower SEE compared to traditional estimation formulas. This model is expected to enable the convenient and reliable estimation of VO2max and the assessment of aerobic capacity in Korean adults using a smartwatch.

## Figures and Tables

**Table 1:** Physical characteristics of the participants.

	**Male (53)**	**Female (51)**	**Total (104)**
Age (years)	35.00 ± 6.11	34.82 ± 7.52	34.91 ± 6.81
Height (cm)	174.09 ± 5.68	161.30 ± 5.33	167.70 ± 8.53
Weight (kg)	74.88 ± 9.95	57.67 ± 7.10	66.44 ± 12.22
BMI (kg/m^2^)	24.71 ± 3.13	22.24 ± 2.66	23.50 ± 3.15
Muscle mass (kg)	34.03 ± 3.69	22.73 ± 2.34	28.49 ± 6.46
Body fat (%)	19.21 ± 6.61	26.91 ± 6.56	22.99 ± 7.61

BMI: body mass index; values are means and ± SD.

**Table 2:** Results of the GXT, Cooper test with a smartwatch, and traditional Cooper test.

	Male (53)	Female (51)	Total (104)
Maximal GXT			
Mean HR (beat/min)	142.44 ± 11.35	139.66 ± 10.57	141.08 ± 11.01
Maximal HR (beat/min)	182.55 ± 13.39	178.22 ± 9.47	180.45 ± 11.80
VO2max (mL/kg/min)	50.06 ± 7.79	42.59 ± 6.38	46.39 ± 8.03
Cooper test with a smartwatch			
Mean HR (beat/min)	164.64 ± 14.80	159.43 ± 21.23	162.09 ± 18.34
Maximal HR (beat/min)	185.89 ± 10.18	181.84 ± 20.13	183.90 ± 15.91
Distance (m)	2676.42 ± 515.49	2276.47 ± 367.92	2480.29 ± 490.13
Traditional Cooper test			
Distance (m)	2683.32 ± 499.40	2264.55 ± 367.21	2477.96 ± 485.41
Estimated VO2max (mL/kg/min)	48.50 ± 11.26	39.31 ± 8.20	43.99 ± 10.86

Values are means and ± SD; GXT: graded exercise test; HR: heart rate.

**Table 3:** Multiple regression model to estimate VO2max using a Cooper test with a smartwatch.

R	R^2^	SEE	F	*p*	Durbin-Watson
0.923	0.853	3.176	93.540	0.000	1.934
	**Unstandardized Coefficients**		**Standardized Coefficients**	**Collinearity Statistic**	
	**B**	**SEE**	**β**	**Tolerance**	**VIF**
Constant	27.620	10.161			
Sex	6.358	1.239	0.398	0.253	3.954
Height (kg)	−0.012	0.064	−0.013	0.332	3.012
Weight (kg)	−0.202	0.046	−0.308	0.306	3.273
Mean HR (beat/min)	−0.036	0.030	−0.082	0.333	3.006
Maximal HR (beat/min)	0.039	0.034	0.078	0.336	2.973
Distance (m)	0.012	0.001	0.730	0.548	1.825

HR: heart rate.

**Table 4:** VO2max prediction model using a Cooper test with a smartwatch and a traditional Cooper test.

	Equation for VO2max	Predicted Value	Measured- Predicted	ICC	CV (%)
Cooper test with a smartwatch	= 27.620 + 6.358 (Sex; male 1, female 0) − 0.012 (Height) − 0.202 (Weight) − 0.036 (Mean HR) + 0.039 (Maximal HR) + 0.012 (Distance, m)	46.48 ± 7.62	−0.09 ± 3.01	0.961	16.39
Traditional Cooper test	= −11.288 + 22.351 (Distance, km)	43.99 ± 10.86	2.40 ± 5.05	0.925	24.69

**Table 5:** Results of simple regression analysis: Cooper test with smartwatch vs. traditional Cooper test.

Tests	B	SE	β	95% CI		R	R^2^	SEE
				Lower	Upper			
Cooper test with a smartwatch								
Intercept	0.973	1.842		−2.680	4.627	0.927	0.860	3.024
Slope	0.977	0.039	0.927	0.900	1.055			
Traditional Cooper test								
Intercept	17.131	1.445		14.266	19.997	0.900	0.810	3.516
Slope	0.665	0.032	0.900	0.649	0.788			8

Values are means and ± SD.

## Data Availability

The data presented in this study are available on request from the corresponding author.
