# Correction: Lee et al. Smartwatch-Derived VO2max Prediction Model for Korean Adults: Utilizing Heart Rate and GPS Data from the 12-Minute Cooper Test. *Healthcare* 2025, *13*, 722

**DOI:** 10.3390/healthcare13101100

**Published:** 2025-05-09

**Authors:** Kihyuk Lee, Dohee Kim, Sungeun Shin, Hongjun Choi, Ahyun Jang, Jinwook Chung

**Affiliations:** 1 Data Convergence Team, Seoul National University Bundang Hospital, Seongnam 13605, Republic of Korea; 2 Department of Sports Science Convergence, College of Arts, Dongguk University, Seoul 04620, Republic of Korea; tara.kim1171@gmail.com (D.K.); mysoulmate1026@gmail.com (S.S.);

There was an error in the original publication [1]. During the manuscript preparation, an incorrect paragraph, unrelated to the Cooper test, was included in Section 2.4. “Cooper 12-Min Test”.

With this correction, the following updated paragraph is being added:

The Cooper test was conducted on a designated 400 m athletic track under the supervision of researchers [9]. Prior to the test, participants completed a 10 min warm-up consisting of continuous running at a low-to-moderate intensity. The test protocol required participants to cover the maximum possible distance within 12 min. Upon completion of the test, the distance traveled was measured using markers placed at 25 m intervals along the track, supplemented by a portable measuring wheel (ML-18MK; Komelon, Korea) for accuracy. During the test, participants wore a Samsung Galaxy Watch 7, which was used to record biometric variables and distance data via PPG and GPS tracking. The recorded biometric variables included the average heart rate (HR), the maximal HR achieved, and the running distance during the 12 min test.

The authors state that the scientific conclusions are unaffected. This correction was approved by the Academic Editor. The original publication has also been updated.
